# Hypoglycemia possibly caused by CYP2C9-mediated drug interaction in combination with bucolome: a case report

**DOI:** 10.1186/s40780-021-00221-y

**Published:** 2021-11-03

**Authors:** Hiroki Tateishi, Daisuke Miyazu, Miho Kurinami, Ichiro Ieiri, Masaaki Hirakawa, Hiroyuki Watanabe

**Affiliations:** 1 Department of Pharmacy, Fukuoka Tokushukai Hospital, 4-5 Sugukita, Kasuga-shi, Fukuoka, 816-0864 Japan; 2 Department of Nutrition, Fukuoka Tokushukai Hospital, 4-5 Sugukita, Kasuga-shi, Fukuoka, 816-0864 Japan; 3 Department of Pharmacy, Kyushu University Hospital, 3-1-1 Maidashi, Higashi-ku, Fukuoka, 812-8582 Japan

**Keywords:** Bucolome, Glimepiride, Hypoglycemia, Drug–drug interaction, CYP2C9

## Abstract

**Background:**

Bucolome is a non-steroidal anti-inflammatory drug and uricosuric agent, currently used only in Japan. It is known to induce drug interactions by inhibiting cytochrome P450 (CYP) 2C9. It is often used to enhance the anticoagulant effect of warfarin by utilizing its drug interactions. There are only a few reports on drug interactions of bucolome and the mechanism remain poorly understood.

**Case presentation:**

An 81-year-old woman with a history of type 2 diabetes mellitus was taking glimepiride 2 mg/day and voglibose 0.6 mg/day. After hospitalization, the patient underwent surgical aortic valve replacement surgery (day 0). Glimepiride and voglibose were resumed on the second postoperative day (day 2), and warfarin was started to prevent thromboembolism. Since the prothrombin time-international normalized ratio on day 9 was low at 1.24, 300 mg/day of bucolome was added to enhance the effect of warfarin. A gradual decrease in blood glucose levels was observed from the day after bucolome administration was initiated. Hypoglycemia in the 56–57 mg/dL range occurred before lunch and dinner on the 6th day (day 14) of bucolome administration, due to which voglibose was discontinued. Hypoglycemia below 70 mg/dL was not observed thereafter, and the general condition of the patient was stable.

**Conclusions:**

Based on the clinical course and literature review, we believe that hypoglycemia in the present case was due to a drug interaction, caused by inhibition of CYP2C9 by bucolome and competitive inhibition of CYP2C9 by warfarin, which affected the pharmacokinetics of glimepiride. The possibility of hypoglycemia due to drug interactions should be considered by physicians, when bucolome is included to enhance the effect of warfarin, in patients taking glimepiride.

## Background

Bucolome is a non-steroidal anti-inflammatory drug and uricosuric agent, used in Japan since 1967. It is known to induce drug interactions by inhibiting cytochrome P450 (CYP) 2C9, and is often used to enhance the anticoagulant effect of warfarin by utilizing such drug interactions [1, 2]. There have been a few clinical reports on bucolome other than in combination with warfarin and on drug interactions due to bucolome. However, the underlying molecular mechanism of its drug interactions remains elusive. Glimepiride is a sulfonylurea antidiabetic drug, and drug interactions via CYP2C9 [3, 4]. Here, we report a case of hypoglycemia after the administration of bucolome to a patient taking glimepiride, a drug for diabetes mellitus, wherein hypoglycemia was speculated to be caused by an interaction between the two drugs. Due consideration was given to the privacy of the patient. This being a case report, the Ethical Review Committee of Fukuoka Tokushukai Hospital waived the requirement of approval by an institutional review board.

## Case presentation

**Fig. 1:**
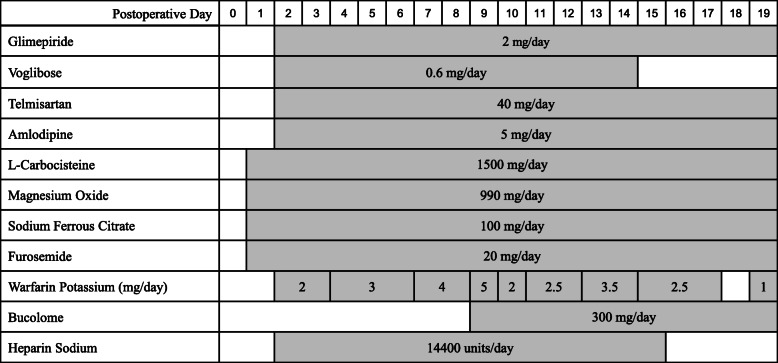
Medications administered postoperatively until discharge. Injectable drugs that whose administration was completed by day 1 are omitted

An 81-year-old woman with type 2 diabetes and hypertension was on glimepiride 2 mg/day (twice a day, after breakfast and dinner), voglibose 0.6 mg/day (three times a day just before meals), telmisartan 40 mg/day (once a day after breakfast), and amlodipine 5 mg/day (once a day after breakfast). The four drugs were taken with good compliance for more than 6 months. Due to exertional dyspnea, she was admitted to our hospital emergency room. Her HbA1c level at admission was well-controlled at 6.1%. Her condition was diagnosed with aortic stenosis, and an aortic valve replacement was performed on an elective basis (day 0). Postoperatively, her respiratory status was good, and her circulatory dynamics were stable without the use of catecholamines. Meals were started on the evening of the first postoperative day. On the same day, ultra-rapid-acting insulin therapy was started using the sliding scale method, based on the pre-prandial blood glucose level. Glimepiride and voglibose were resumed on the second postoperative day, and warfarin was included to prevent thromboembolism. Warfarin was dose-adjusted using the prothrombin time-international normalized ratio (PT-INR). Since the PT-INR was low (1.24) on postoperative day 9, 300 mg/day of bucolome was started to enhance the effect of warfarin. The medications used post-surgery are shown in Fig. 1, and the changes in laboratory values are shown in Table 1.

**Table 1:** Laboratory values of the patient

		Postoperative day							
		1	3	5	7	10	13	15	18
AST	U/L	32	20	21	23	17	14	20	18
ALT	U/L	8	7	10	13	12	10	13	13
LDH	U/L	358	277	248	285	271	223	241	203
Alb	g/dL	3.8	3.1	2.9	3.0	3.3	3.2	3.4	3.6
BUN	mg/dL	13.1	19.2	12.5	7.9	8.0	6.1	5.7	10.5
Cre	mg/dL	0.78	0.86	0.58	0.62	0.72	0.68	0.66	0.77

*AST* aspartate transaminase, *ALT* alanine transaminase, *LDH* lactate dehydrogenase, *Alb* albumin, *BUN* blood urea nitrogen, *Cre* creatinine

**Fig. 2:**
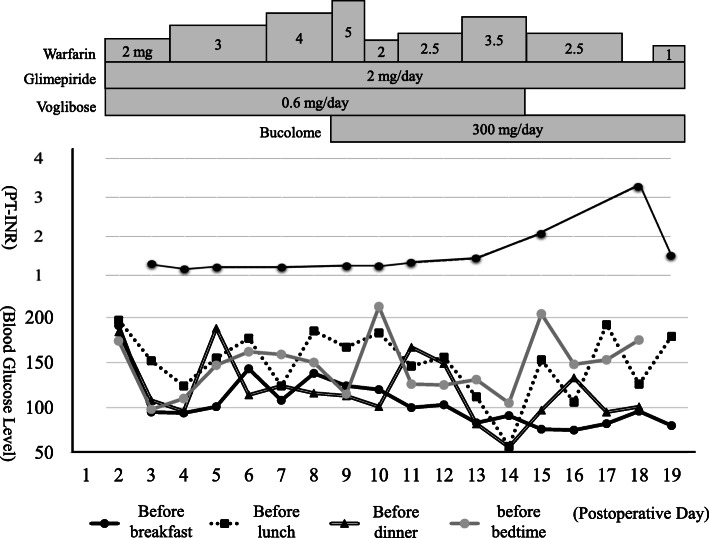
Clinical course of the patient. Doses of warfarin, glimepiride, voglibose, and bucolome. PT-INR and blood glucose level trends from the postoperative period to the day of discharge.PT-INR: prothrombin time-international normalized ratio

The PT-INR began to increase after bucolome administration was initiated, and a decrease in blood glucose level was observed from day 2 of bucolome administration. On day 6 of bucolome administration, the patient experienced hypoglycemia in the range of 56–57 mg/dL before lunch and dinner. We suggested the possibility of drug-induced hypoglycemia associated with the administration of bucolome, and asked the physician to reduce the dose of antidiabetic drugs. Voglibose was discontinued the next day and increased blood glucose levels were observed before lunch, dinner, and bedtime. Contrastingly, the blood glucose levels before breakfast remained unchanged at 70–90 mg/dL. The trends in blood glucose levels are shown in Fig. 2. Thereafter, the patient was transferred to another hospital because hypoglycemia below 70 mg/dL was not observed and her general condition was stable (day 19). Information regarding hypoglycemia and PT-INR fluctuation during the initial hospitalization was provided to the transfer hospital by documenting it in a medication notebook. A special diet for patients with diabetes (1600 kcal/day) was provided during hospitalization and the food intake was consistent throughout. Apart from meals, maintenance fluid (20 mL/h, 3.4 kcal/h) was administered through peripheral vessels from postoperative day 1 to postoperative day 15. No significant changes in liver and kidney function or albumin levels were observed. Furthermore, the pharmacist confirmed that there was no drug overdose or intake of supplements.

## Discussion

A gradual decrease in blood glucose level of the patient was observed with the administration of bucolome; hypoglycemia occurred on the 6th day of bucolome administration. There are no reports on hypoglycemia as a side effect of Bucolome [5]. We suspected drug-induced hypoglycemia indirectly related to bucolome, because the patient’s food intake was consistent during hospitalization and her general condition remained stable without any postoperative complication such as infections.

Glimepiride acts on pancreatic β-cells to promote insulin secretion, irrespective of the blood sugar levels. The half-life of glimepiride is 3–4 h, but the effect lasts for more than 24 h [6]. The unchanged form of glimepiride is the main pharmacologically active form. It is metabolized by CYP2C9, and the main metabolite has approximately one-third of the pharmacological activity of the unchanged form [6]. It has been reported that in patients taking glimepiride, the dose of glimepiride was significantly lower in the concomitant use group of typical CYP2C9 inhibitors such as metronidazole and fluconazole [3]. In addition, it has been reported that the area under curve of glimepiride was significantly decreased in the concomitant use group of rifampicin, which induces CYP2C9 [4]. From these reports it can be inferred that the concomitant use of drugs which are either CYP2C9 inducers or inhibitors, may cause changes in the blood concentration of glimepiride. Bucolome inhibits the metabolic activity of CYP2C9 and its concomitant use can increase the blood concentration of losartan, a typical substrate of CYP2C9 [1, 7]. These reports suggest that bucolome, a CYP2C9 inhibitor, may increase the blood concentration of glimepiride by inhibiting the metabolism of glimepiride, a substrate of CYP2C9. In the present case, hypoglycemia was observed on the next day after bucolome administration was initiated, and hypoglycemia in the range of 50 mg/dL occurred on the 6th day of bucolome administration. Since the biological half-life of bucolome is approximately 29 h [5], it takes time to reach a steady state. We believe that the inhibitory effect of bucolome on CYP2C9 increases gradually, while the hypoglycemic effect of glimepiride also simultaneously increases. Furthermore, the changes in blood glucose levels after bucolome administration suggest that bucolome can inhibit the metabolism of glimepiride.

Herein, we must also consider the effects of warfarin. Warfarin is a drug that exhibits anticoagulant activity by acting on vitamin K epoxide reductase and inhibiting the production of coagulation factors. Warfarin is a racemic drug, and its S-form is three to five times more pharmacologically active than its R-form [8]. S-warfarin is mainly metabolized by CYP2C9, whereas the R-form is mainly metabolized by CYP3A4 and CYP1A2 [9]. Inhibition of CYP2C9, a metabolic enzyme that greatly influences the effect of warfarin, by bucolome causes an increase in the blood concentration of warfarin [10]. In this case, the PT-INR was high after the administration of bucolome, whereas it was not high when warfarin was administered alone. Interestingly, the blood glucose levels also decreased. It has been reported that the rate of hospitalization or frequency of emergency room visits due to hypoglycemia, was higher in the sulfonylurea and warfarin combination group than in the noncombination group [11]. The mechanism of hypoglycemia here, may be the competition for metabolism by CYP2C9; since sulfonylurea and warfarin are both primarily metabolized by CYP2C9, higher doses of warfarin may limit the rate at which sulfonylurea can be metabolized. This suggests that warfarin may enhance the hypoglycemic effect of glimepiride by inhibiting its metabolism via CYP2C9. It has also been reported that warfarin itself may have a hypoglycemic effect [12]. Since bucolome increases the blood concentration of warfarin [10], warfarin can possibly increase the risk of developing hypoglycemia. Furthermore, it has been reported that glimepiride competitively inhibits warfarin metabolism via CYP2C9 in an in vitro system [13]. Although there is a lack of knowledge on this subject, it is possible that both, glimepiride, a common substrate of CYP2C9, as well as bucolome, may have influenced the PT-INR changes in this case. When multiple CYP2C9 substrates are included in the treatment regimen, such as warfarin and glimepiride, it is necessary to consider the possibility that they may interact with each other.

In addition to the mechanism of inhibition of CYP2C9, interaction with serum protein via binding and/or substitution should also be considered. Warfarin, bucolome, and glimepiride have a high serum protein-binding capacity and can bind to albumin [8, 14, 15]. Warfarin and bucolome bind to binding site I, and glimepiride to binding sites I and II of albumin [16–18]. Therefore, it is possible that the binding displacement at binding site I may influence the effect of glimepiride. However, because many of the drug interactions due to serum protein-binding displacement are thought to have little effect on the concentration of the free drug in the blood for orally administered drugs [19–21], the drug interactions are unlikely to be the cause of hypoglycemia in this case.

Voglibose and glimepiride have been reported to cause hypoglycemia, among the medications that the patient was taking. Voglibose is an α-glucosidase inhibitor that improves postprandial hyperglycemia. Voglibose hardly migrates into the blood, shows a direct medicinal effect in the intestinal tract, and is excreted in the feces [22]. Based on the pharmacological mechanism, it is unlikely that an α-glucosidase inhibitor alone can induce hypoglycemia. The incidence of hypoglycemia with voglibose alone is lower than that with other antidiabetic drugs [23, 24]. In this case, there were no complications, such as other diseases and infections, and the patient was consuming food without any problems. Although the hypoglycemia improved with the discontinuation of voglibose, it was not considered to be the direct cause of hypoglycemia. On the contrary, discontinuation of voglibose on the day after the onset of hypoglycemia caused an increase in blood glucose levels before lunch, dinner, and bedtime. This may be because voglibose suppressed the absorption of some dietary sugars in the small intestine, but when voglibose administration was discontinued, dietary sugars were absorbed in the small intestine and remained unmetabolized until the time of the next meal. In contrast, the early morning blood glucose level continued to decrease, regardless of voglibose discontinuation. This may be because voglibose has little effect on the early morning blood glucose level, while the effect of glimepiride continues.

Based on the clinical course and literature review, we believe that the hypoglycemia in this case was due to a drug interaction, caused by inhibition of CYP2C9 by bucolome and competitive inhibition of CYP2C9 by warfarin, which affected the pharmacokinetics of glimepiride. Since, the duration from the occurrence of hypoglycemia to discharge was short, there was insufficient time to adjust the dosage of the antidiabetic drug. Although the discontinuation of voglibose prevented the patient from developing hypoglycemia (blood glucose levels below 70 mg/dL), glimepiride dosage should have been reduced or discontinued in consideration of the risk of hypoglycemia in the future. Pharmacists are required to foresee the occurrence of rare drug interactions from a pharmaceutical point of view, as in this case. In other words, it is important to provide information to physicians and nurses on the risk of hypoglycemia caused by the combination of bucolome and glimepiride and instruct patients on how to cope with the occurrence of hypoglycemia, for the safe management of drug therapy.

In clinical practice in Japan, bucolome is mostly used to enhance the effect of warfarin; therefore, we could not investigate the effect of bucolome alone on glimepiride. In addition, we suspected a drug interaction based only on blood glucose level trends; however, it is necessary to measure the blood concentration of glimepiride for a more accurate investigation. Additionally, genetic polymorphisms of CYP2C9 in the Japanese population, has been reported [25]; however, we were unable to determine the genotype of the patient. Therefore, it is necessary to accumulate similar cases, stratify them according to pharmacokinetics and pharmacodynamics, and analyze each process based on genomic pharmacology, to generate an appropriate design/strategy for drug use and safety information.

In conclusion, we have reported a case of drug-induced hypoglycemia due to glimepiride, which may have been caused by the concomitant use of bucolome. The possibility of hypoglycemia due to drug interactions should be considered when bucolome is used to enhance the effect of warfarin, in patients taking glimepiride.

## Data Availability

The dataset generated and analyzed in this case report will not be shared due to the risk of identifying the patient.

## Abbreviations

CYP: Cytochrome P450
PT-INR: Prothrombin time-international normalized ratio
